# Detection of EGFR gene mutations in non-small cell lung cancer: Lessons from a single-institution routine analysis of 1,403 tumor samples

**DOI:** 10.3892/ijo.2013.2056

**Published:** 2013-08-07

**Authors:** AUDREY VALLEE, CHRISTINE SAGAN, ANNE-GAELLE LE LOUPP, KALYANE BACH, THOMAS DEJOIE, MARC G. DENIS

**Affiliations:** 1 Departments of Biochemistry, Nantes University Hospital, Nantes, France; 2 Pathology, Nantes University Hospital, Nantes, France

**Keywords:** non-small cell lung cancer, epidermal growth factor receptor mutation, TTF-1 expression

## Abstract

Activating mutations of the epidermal growth factor receptor (EGFR) in lung tumors are associated with a dramatic response to tyrosine kinase inhibitors. Therefore, routine analysis of pathological specimens is mandatory in clinical practice. We have prospectively tested tumors from Caucasian lung tumor patients between January 2010 and June 2012. DNA was extracted from formalin-fixed paraffin-embedded tissues following macrodissection. The p.L858R substitution was assessed by allele-specific PCR and exon 19 deletions by PCR and DNA fragment analysis. Using a robust process from patient sampling to screening methods, we analyzed samples from 1,403 patients. The EGFR status could be successfully determined for 1,322 patients. EGFR mutations were detected in 179 (13.5%) patients, with female and adenocarcinoma histology predominance. Mutated patients were significantly older than non-mutated patients. Similar mutation rates were obtained with primary tumors and metastases, and with surgical resection, bronchial biopsies, CT-guided needle biopsies and transbronchial needle aspiration. The sensitivity of our assays allowed us to detect EGFR mutations in samples poor (<10%) in tumor cells. Finally, the mutation rate was much higher in tumors expressing the TTF-1 antigen (145/820; 17.7%) than in TTF-1 negative tumors (3/218; 1.4%). The results obtained through routine analysis of more than 1,300 samples indicated that all types of specimen can be analyzed without any significant bias. TTF-1 immunostaining may be used to predict negative EGFR mutation status.

## Introduction

Prospective randomized clinical trials have shown that tyrosine kinase inhibitors (TKI) gefitinib ( 1 – 3 ) and erlotinib ( 4 , 5 ) as initial treatment for EGFR mutation-positive advanced NSCLC improved outcomes compared with chemotherapy. These molecules have thus been approved in many countries worldwide. Therefore, routine analysis of pathological specimens is mandatory in clinical practice to predict patient response. The potential result is an increased likelihood that patients will receive optimal therapy for their tumour and be spared a course of therapy with no or significantly less benefit. For that promise to be realized, a robust process, from patient sampling to screening methods, has to be developed to be capable of fast, reliable, sensitive and reproducible detection of the mutations in patient tumor samples.

In current clinical practice, the samples available for detection of somatic mutations are most of the time formalin-fixed paraffin-embedded tissues of various tumor sites. The samples are usually composed of mutant and wild-type DNA from tumor cells and wild-type DNA from non-malignant cells (normal epithelial cells, hematopoietic cells and stromal cells such as fibroblasts). Therefore there is a need for a sensitive technique and a complete reliable process. If standard dideoxy sequencing has been the ‘gold standard’ for detecting mutations in constitutive genetics, this robust method is however time-consuming, has only moderate sensitivity and might suffer from a lack of robustness when working on fragmented DNA extracted from formalin fixed paraffin embedded tumors ( 6 , 7 ) . These limitations of direct sequencing for detecting somatic mutations has led to the development of more sensitive, less expensive, and faster methods. A number of alternative procedures have therefore been developed to detect common cancer mutations, such as HRM ( 8 – 10 ) , allele-specific amplification ( 11 , 12 ) , primer extension ( 13 ) , and pyrosequencing ( 14 ) . In most cases, a better sensitivity was obtained using targeted techniques as compared to direct sequencing ( 15 , 16 ) ; reviewed in Ellison *et al* ( 17 ) .

We developed assays aiming at accurately detecting EGFR mutations in patient tumor samples in routine screening. The assays had to detect exon 19 deletions and the p.L858R (exon 21) mutations, the two most common mutations in NSCLC that are clearly associated with a clinical benefit. These assays, fragment analysis (exon 19) and allele specific PCR (L858R) have been routinely used for the last 3 years in our laboratory. Moreover, during this period, we collected information on the patients (age, gender) and the samples tested: histology, thyroid transcription factor-1 (TTF-1) expression, primary or metastatic lesion, type of specimen, and tumor cell content. We undertook the analysis of the data obtained. This allowed us to evaluate the impact of these parameters on the frequency and spectrum of EGFR mutations in Caucasian NSCLC patients. Here we report our experience testing for EGFR mutations in a large number of samples using sensitive techniques in a clinical setting.

## Materials and methods

### Patients

A total of 1,403 formalin-fixed paraffin-embedded tumor samples from NSCLC patients were referred to our laboratory for EGFR typing between January 2010 and June 2012. There were 1,243 adenocarcinomas, 49 squamous cell carcinomas and 111 non-small cell carcinomas, from 827 men and 576 women.

### Sample processing and DNA extraction

Serial sections were cut from each paraffin block. Tumor-rich areas were marked by the pathologist on a hematoxylin and eosin 3 *μ* m-thick stained section. To eliminate non-malignant, stromal and contaminating inflammatory cells and to enrich the analyzed specimen with tumor cells, these areas were manually macro-dissected on 10 *μ* m-thick sections using single-use sterilized scalpels. DNA was then extracted after paraffin removal (toluene 5 min, ethanol 3 min, ethanol 2 min) using the Forensic kit and an iPrep system according to the manufacturer’s recommendations (Invitrogen, Life Technologies SAS, Villebon sur Yvette, France). DNA concentration was quantified by spectro-photometry (NanoDrop ND-100 instrument, Thermo Fisher Scientific, Waltham, MA) and normalized to 5 ng/ *μ* l.

### Detection of the L858R mutation - allele specific amplification

**Figure 1.**
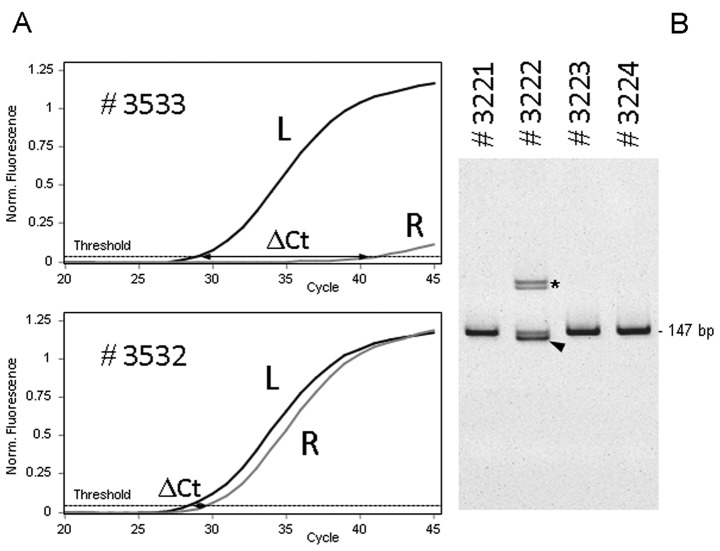
Molecular analysis of EGFR mutations. (A) Representative amplification plots for a tumor presenting a p.L858R mutation (no. 3532, ΔCt=1.0) or wild-type for this allele (no. 3533, ΔCt=12.6). (B) Polyacrylamide gel electrophoresis showing several tumors presenting a wt exon 19 (nos. 3221, 3223 and 3224), and tumor no. 3222 presenting an exon 19 deletion. The arrowhead indicates the deleted amplicon, and the star shows the heteroduplexes. Methodological details are described in Materials and methods.

For codon 858 mutation, we designed two forward primers with variations in their 3′ nucleotides such that each was specific for the wild-type (858L; TCAAGATCACAGATTT TGGGCT) or the mutated variant (858R; TCAAGATCACAG ATTTTGGGCG), and one common reverse primer (AS; CATC CTCCCCTGCATGTGTTAAAC). The sequence-specific forward and the reverse primer were then combined in ‘Primer mix L’ (primers 858L and AS), and ‘Primer mix R’ (primers 858R and AS). The amplification conditions were optimized for the RotorGene 3000 instrument (Qiagen, Courtaboeuf, France). PCR amplifications were performed using the LC480 SYBR-Green mix (Roche Diagnostics, Meylan, France). The reaction mixture contained 10 *μ* l of the supplied 2X master mix, 0.5 *μ* l of each primer (10 *μ* M each), and 9 *μ* l of the template (45 ng genomic DNA). The cycling conditions were as follows: denaturation for 10 min at 95°C; amplification for 45 cycles, with denaturation for 10 sec at 95°C, annealing for 15 sec at 65°C, and extension for 20 sec at 72°C. The specific 172-bp PCR products were amplified, and the cycle threshold (Ct)-value was determined for a fixed normalized fluorescence of 0.05. For each sample, the Ct-value was determined for the 858L (control) PCR, and for the 858R (mutation specific) PCR, and the difference was calculated (ΔCt, Fig. 1A ). The lower the amount of mutated DNA in the sample, the higher the ΔCt value. Samples with ΔCt<5 were considered as positive for the p.L858R mutation. Fig. 1A illustrates the assay performed on formalin-fixed paraffin-embedded extracted DNAs containing a p.L858R mutation (#3532, ΔCt=1.0) or wild-type for this allele (#3533, ΔCt=12.6).

### Detection of exon 19 deletions - fragment size analysis

PCR were performed as described above using primers 19S (GTCT TCCTTCTCTCTCTGTCATAG) and 19AS (CCACACAGCA AAGCAGAAACTCAC). Following amplification, amplicons were analyzed by polyacrylamide gel electrophoresis on 5-20% acrylamide gels (Invitrogen, Life Technologies, Saint Aubin, France). Migration was performed for 1 h at 100 V. The wild-type EGFR gene yielded a 147-bp amplicon. Deletions were identified as faster migrating bands. Fig. 1B illustrates the profiles obtained for exon 19 wild-type tumor (#3221, #3223 and #3224) and a tumor presenting an exon 19 deletion (#3222). In case of exon 19 deletions, a doublet (or in rare cases a single band) was seen at higher molecular weight. We have previously described that each deletion is characterized by specific additional bands corresponding to heteroduplexes ( 18 ) .

### Statistical analysis

The χ ^2^ test, the Fisher’s exact test and the two-tailed non-parametric Mann-Whitney test were used to assess the association between mutation status of EGFR and each of the clinicopathological parameters. A p-value of 0.05 was considered statistically significant.

## Results

### Assay performance

The sensitivity and specificity of our assay were evaluated for the specified EGFR alteration in quality control schemes.

First, serial dilutions of DNA extracted from cell lines harboring a p.L858R mutation (NCI-H1975: p.L858R; c.2573T>G) or an exon 19 deletion (NCI-H1650: p.E746- A750del; c.2235-2249del) in wild-type DNA were analyzed. This revealed that our techniques allowed us to detect an EGFR alteration when it is present in at least 1% (p.L858R mutation) or 2% of cells (exon 19 deletions). However, DNA extracted from FFPE samples is of much lower quality than DNA extracted from cell lines. Therefore, in order to avoid false negative results, we considered that a minimum of 10% of tumor cells should be present in the sample. If no alteration was found in a sample presenting less than 10% of tumor cells, we concluded that the test was not contributive.

To assess the specificity of our assays, we repeated the analysis of a large number of samples using an approved kit (Therascreen EGFR RGQ kit, Qiagen, Hilden, Germany). We tested 160 samples that did not present a mutation on exon 19 and 21, and 98 samples presenting an exon 19 deletion or the L858R point mutation. Identical results were obtained for all these samples.

Finally, our laboratory has been involved in external quality schemes organized in western France in 2010 and 2011. Twenty NSCLC samples were tested during this period and we found concordant results with the 5 other centers in all these cases. More recently we have also been involved in the first external quality control scheme organized by the French National Cancer Institute. Sections from 10 NSCLC specimens were sent to all the laboratories performing EGFR testing in France. We obtained the expected result for all these samples using our procedures.

In routine practice, FFPE sections were sent from the department of pathology to the department of molecular biology every wednesday, and the reports were sent to the oncologists the next friday. The molecular biology laboratory turnaround time is thus of 48 h.

### Frequency and spectrum of EGFR mutations

**Figure 2.**
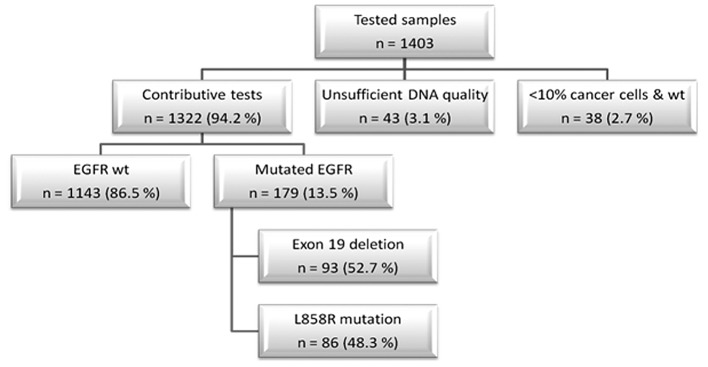
NSCLC samples submitted to EGFR testing in our hospital between January 2010 and June 2012.

Our study population was composed of 1,403 NSCLC patients (417 females and 725 males; median age 64). The tests failed in 43 cases (3.1%), because of insufficient DNA quality ( Fig. 2 ). In 38 cases (2.7%) where we were unable to detect an EGFR alteration, the content of tumor cells in the sample was considered too low to conclude. The remaining 1,322 tests performed (94.2%) were contributive. We were able to identify an EGFR alteration in 179 patients (13.5%). These included 93 exon 19 deletions (52.7% of the detected alterations), and 86 L858R point mutations (48.3%) ( Fig. 2 ).

The clinicopathologic features of patients with EGFR- mutated or wild-type tumors in our cohort are summa rized in Table I . Consistent with previous observations, the mutation rate was significantly higher in women than in men (23.0 vs 6.9%), and mutations were more frequent in adenocarcinomas (168/1,144; 14.6%) than in other histological types. Interestingly, EGFR-mutated patients were significantly older than those with wild-type EGFR (median age 71 vs 63 years; p<10 ^−4^ ).

Recent data reported by Sun *et al* ( 19 ) showed a correlation between TTF-1 expression and EGFR mutation status. Thus we also analyzed TTF-1 expression in our series of patients. Our data also clearly indicated that EGFR mutations are more likely to occur in TTF-1 positive tumors (145/675; 17.7%) than in TTF-1 negative adenocarcinomas (3/218; 1.4%). Almost all (145/148; 98.0%) mutated adenocarcinomas were TTF-1 positive ( Table II ).

We next addressed the influence of tumor site and type of sampling ( Table III ). The frequency of EGFR alteration was found to be slightly higher in metastases than in primary tumors (16.2 vs 13.5%, respectively), but it was not statistically different (p=0.27). Considering primary tumors, we did not see a significant difference between surgical specimen (35/294; 11.9%), bronchial biopsies (42/339; 12.4%) and transthoracic needle biopsies (37/258; 14.3%). When considering metastases, we also observed similar mutation rate when analyzing biopsies (23/183; 12.6%), surgical resection (20/135; 14.8%), and transbronchial needle aspirates (5/38; 13.2%). The mutation rate seemed to be higher in pleural effusions (11/44; 25.0%), but it was not statistically significant.

Finally, we analyzed our data according to the percentage of cancer cells present in the samples tested. Similar mutation rates were obtained, even with samples containing less than 10% of cancer cells ( Table IV ).

## Discussion

EGFR is a target of the TKIs gefitinib and erlotinib, which have been approved for advanced NSCLC treatment in many countries. Various EGFR testing approaches have been used in the different phase III trials that led to these approvals. The Scorpion Amplification Refractory Mutation System (ARMS) was used in the phase III Iressa Pan-Asia Study (IPASS) to determine *EGFR* mutation status ( 1 ) . A variety of methods, including direct sequencing, PCR-invader, PNA-LNA PCR clamp, fragment analysis, and cycleave PCR, were used in the WJTOG3405 phase III study to select *EGFR* mutation-positive patients ( 2 ) , and the PNA-LNA PCR clamp method was used in the NEJ002 study ( 3 ) . In the European EURTAC study, tissue samples were analyzed with Sanger sequencing (exons 19 and 21), and EGFR mutations were confirmed with an independent technique (deletions in exon 19 by length analysis and L858R mutations in exon 21 were detected with a 5′ nuclease PCR assay) ( 5 ) . Finally, in the Optimal trial, testing was done by PCR-based direct sequencing, and other methods were applied for monitoring at the same time (gel electrophoresis for *EGFR* exon 19 deletions and cycleave real-time PCR for *EGFR* exon 21 L858R mutations) ( 4 ) .

Several years ago, we developed a sensitive procedure for routine analysis of NSCLC tumors, and the aim of our study was to evaluate its performance through analysis of the results obtained during a long period. Between January 2010 and June 2012, we tested 1,403 tumors in a routine clinical setting. The tests failed in only 3.1% of the samples tested because of insufficient quality of DNA. These not contributive tests were not associated with the type or the size of the samples tested. But this rate was significantly higher in samples processed in 2 of the pathology centers that sent samples to our platform (not shown). We are at present trying to identify the cause(s) of the lower quality of DNA in some tissues processed in these centers. When excluding these centers from the analysis, the failure rate decreased down to 1% (12/1,194 samples).

We found that 179 of the tumors tested harbored an exon 19 deletion or an L858R point mutation. This corresponded to 13.5% of the 1,322 contributive samples. These findings are in keeping with previous reports on Caucasian patients. For instance, molecular testing of 755 patients from UK revealed a mutation prevalence of 13% ( 20 ) , and 13.1% of Portuguese patients were found to present an EGFR mutation ( 21 ) . A large analysis of Spanish patients revealed a slightly higher mutation rate (16.6%), but the authors hypothesized that the participating centers included more samples from women and patients who had never smoked ( 22 ) .

In 2004, several groups correlated responses to gefitinib or erlotinib with the presence of somatic mutations clustered around the ATP-binding pocket of the tyrosine kinase domain ( 23 , 24 ) . Subsequent reports indicated that the somatic mutational status of EGFR correlated with female sex ( 25 , 26 ) , smoking history ( 25 , 27 , 28 ) , adenocarcinoma histology ( 26 , 29 ) and Asian origin ( 26 ) .

We were not able to collect the smoking history of the patients tested during this period in our platform, but the mutation rate was clearly associated with female sex and adenocarcinoma histology. Moreover, patients with a mutation were clearly older than wild-type patients, as previously described for patients from Japan ( 30 ) and Korea ( 31 ) . In addition, we clearly demonstrated that the overwhelming majority of EGFR mutated tumors expressed the TTF-1 antigen.

In a recent report, Girard *et al* collected clinical and pathological data on a large number of patients with NSCLC who had their tumors genotyped for *EGFR* mutations at different institutions. Variables of interest (smoking history, histological subtype, sex, stage of disease and age) were integrated in a multivariate logistic regression model, and they could thus build a model-based nomogram to allow for prediction of the presence of *EGFR* mutations in NSCLC ( 32 ) . Unfortunately, TTF-1 expression data were lacking, and they could not include this covariate in the final model.

In our series, the EGFR mutation prevalence was clearly higher for women with a TTF-1 positive adenocarcinoma (28.3%; 101/357). The mutation prevalence increased to 46.7% (64/137) when selecting women older than 70.

Our analysis of this large series of patients indicated that the mutation rate was not significantly different between primary tumors and metastases. More importantly, we demonstrated that there is no impact of the type of specimen tested on the mutation prevalence. Similar levels of contributive results (not shown) and of EGFR mutation rates were obtained using different type of samples, including pleural fluids and trans-bronchial needle aspirates.

Finally, we also found, using a sensitive approach, that the mutation rate was not dependent on the sample cellularity. Indeed, 23 patients for whom the tested samples contained less than 25% of cancer cells (<10 and 10–25%) presented an EGFR mutation (23/170; 13.5%). Using a less sensitive technique such as direct sequencing, we would have most likely missed these mutations. Consequently, these patients would have been treated by chemotherapy, and not benefited from TKI. That is the reason why, in routine diagnosis, we do not reject samples containing less than 10% tumor cells. In a recent report Warth *et al* described an algorithm for Sanger sequencing-based EGFR mutation analyses ( 33 ) . They demonstrated that sequencing generated 80% reliable analysis of biopsy specimens, but that in 20% of cases rebiopsy had to be recommended. A more sensitive technique such as the one we use on a routine practice avoids performing additional biopsies.

The clinical impact of detecting mutations present in a low percentage of tumor cells has been assessed by Zhou *et al* ( 34 ) . They determined whether abundance of *EGFR* mutations in tumors predicts benefit from treatment with EGFR-TKIs for advanced NSCLC. They detected EGFR mutations in lung cancer samples using both direct DNA sequencing and the sensitive ARMS technique. Mutation-positive tumors by both methods carried high abundance of EGFR mutations, and tumors that were mutation positive by ARMS but mutation negative by direct DNA sequencing harbored low abundance of EGFR mutations. Median progression free survival of patients with low abundance of EGFR mutations was significantly longer than for those with wild-type tumors, and the difference between patients with high and low abundance of EGFR mutations was not significant regarding overall response rate and overall survival. Thus, it is worth using a sensitive technique to detect patients with a low abundance of mutations.

In conclusion, we demonstrated that performing EGFR testing in routine diagnostic and clinical practice using sensitive approaches can be successful. In our French population, the EGFR mutation prevalence is higher in older patients, women, adenocarcinomas and TTF-1 expressing adenocarcinomas.

## Figures and Tables

**Table I.** Clinical and pathological characteristics associated with EGFR mutational status in 1,322 french patients with lung tumors.

	Total	EGFR wt	EGFR mutated	P-value
Gender				
Female	543	418 (77.0%)	125 (23.0%)	
Male	779	725 (93.1%)	54 (6.9%)	<0.0001
Age, years				
Median		63	71	<0.0001
Range		28–100	32–92	
Histology				
Adenocarcinoma	1,144	976	168 (14.7%)	
NSCLC-NOS	101	97	4 (4.0%)	0.004
Squamous cell	45	42	3 (6.7%)	

**Table II.** Relationship between TTF-1 immunostaining and EGFR mutation frequency in adenocarcinomas.

	Total	EGFR wt	EGFR mutated	P-value
TTF-1				
IHC ^ + ^	820	675 (82.3%)	145 (17.7%)	
IHC ^ − ^	218	215 (98.6%)	3 (1.4%)	<0.0001

**Table III.** Influence of tumor site and type of sample on the EGFR mutation frequency.

	Total	EGFR wt	EGFR mutated
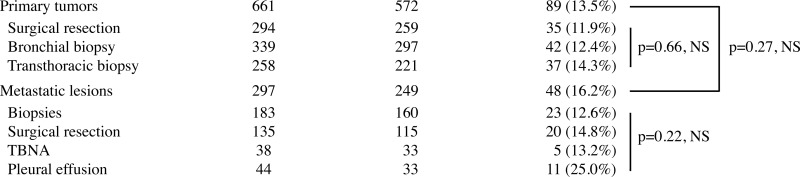			

NS, non-significant.

**Table IV.** Influence of the cellularity of tested samples on the EGFR mutation frequency.

	Total	EGFR wt	EGFR mutated	
Tumor cell content				
>50%	669	590 (88.2%)	79 (11.8%)	
25–50%	477	407 (85.3%)	70 (14.7%)	p=0.46, NS
10–25%	156	138 (85.5%)	18 (11.5%)	
<10%	45	NC (84.4%) ^a^	7 (15.6%)	

a If no alteration was found in a sample presenting less than 10% of tumor cells, we concluded that the test was not contributive (NC). NS, non-significant.
